# Hazard identification and the prevalence of occupational skin disease in Indonesian Batik workers

**DOI:** 10.1038/s41598-022-17890-w

**Published:** 2023-03-30

**Authors:** Sri Awalia Febriana, Yohanes Ridora, Niken Indrastuti, Kusuma Dewi, Erdina, Katharina Oginawati, Ikeu Tanziha, Cita Rosita Sigit Prakoeswa, Fajar Waskito, Marie-Louise Schuttelaar

**Affiliations:** 1 Department of Dermatology and Venereology Faculty of Medicine, Public Health, and Nursing, Universitas Gadjah Mada/Dr. Sardjito Hospital, Farmako Street, Sekip Utara, 55281 Yogyakarta, Indonesia; 2 Faculty of Civil Engineering and Environment, Institut Teknologi Bandung, Bandung, Indonesia; 3 Faculty of Human Ecology, IPB University, Bogor, Indonesia; 4 Faculty of Medicine, Universitas Airlangga/Dr. Soetomo General Academic Hospital, Surabaya, Indonesia; 5 Department of Dermatology, University Medical Centre Groningen/the University of Groningen, Groningen, The Netherlands

**Keywords:** Health care, Health occupations, Diseases, Skin diseases

## Abstract

Batik, a resist-dyeing technique to decorate a special cotton fabric, has been practiced for centuries in Indonesia. Unfortunately, as an informal enterprise, batik industry activities lack work safety and health regulations. This study aimed to identify potential health hazards, including inventorying the chemicals to which the workers are exposed, the PPE profile, and investigating the prevalence of occupational skin diseases (OSD) in the batik industry. A cross-sectional study and an inventory of exposure to the chemicals were done in traditional batik workplaces in 5 districts in Yogyakarta province, Indonesia. The chemicals were classified as potential sensitizers/irritants, and the workers were examined and interviewed using the Nordic Occupational Skin Questionnaire-2002/LONG. Of 222 traditional batik workers, OSD were diagnosed in 61 (27.5%) workers, with occupational contact dermatitis was the most common OSD encountered (n = 23/61; 37.7%) (allergic contact dermatitis n = 7/23; irritant contact dermatitis n = 16/23). A smaller portion of other OSD was also encountered including callus, miliaria, and nail disorder (9%, 6.3%, and 5.9%, respectively). During each step of the traditional batik manufacturing process, the workers are exposed to substances that act as irritants and/or as potential contact allergens. However, only one-fourth of the workers used PPE regularly, particularly during the coloring process and wax removal (wet processes). Traditional batik manufacturing process exposes the worker to various physical and chemical hazards, resulting in a high prevalence of occupational skin diseases, especially contact dermatitis among the employees.

## Introduction

Batik is part of the Indonesian cultural heritage which has been widely recognized in various parts of the world. In 2009, UNESCO designated Indonesian batik as a Masterpiece of Oral and Intangible Heritage of Humanity. Batik is currently one of the sources of livelihood in Indonesia. Yogyakarta was appointed as Batik City in 2014 by the World Craft Council (WCC). According to Dekranasda DIY, the national board of crafts in Yogyakarta, it is estimated there are as many as 8000 Batik Community Activities Units in Yogyakarta in 2016^1^.

Batik is a technique to decorate a special cotton fabric by applying a resist-dyeing technique that has been practiced for centuries in Java, Indonesia, as a part of an ancient tradition. In brief, it begins with processing the cotton fabrics, followed by wax-painting with traditional batik wax which mainly contains paraffin and beeswax, then the coloring process, and ends with removing the remaining wax^2^. Batik is made by brushing or drawing hot wax over the cloth and followed by dyeing the cloth. The part which is covered by wax resists the dye and leaves the original color unchanged. To make a more elaborate and colorful design, the process of waxing and dyeing can be repeated. All these procedures are done in a home industry setting without using factory machinery^2^.

Unfortunately, as an informal enterprise, batik industry activities lack work safety and health regulations. One of the most common problems encountered is the minimal use of Personal Protective Equipment (PPE) by the workers during the processes in which the skin is exposed to substances that pose a risk of causing skin diseases at work^3^. Occupational skin diseases (OSD is the second most common occupational ailment with a prevalence of around 29%, following musculoskeletal disorder)^4^. Occupational contact dermatitis (OCD) is an inflammatory skin disorder caused by work-related exposure to irritants or contact allergens^5^, and accounts for 90–95% of all OSD^6^.

The batik process exposes the workers to various physical and chemical hazards, including skin irritants and contact allergens, which may result in developing skin disorders. Several studies have been conducted regarding the prevalence of occupational skin diseases in traditional batik manufacturing workers, but information related to the characteristics of chemicals involved and patch test results are still limited. A study conducted by Kusbandono et al. showed a prevalence of OSD among batik manufacturing workers in Yogyakarta of 28.13%, of which 41.6% were OCD^7^. The purpose of the current study is to describe details of the work process in the batik industry and to identify potential health hazards including making an inventory of the chemicals to which the workers are exposed and the use of PPE among batik workers. We also aim to investigate the prevalence of occupational skin diseases in the batik industry. Contact sensitization among these batik workers has been described in a separate paper^8^.

## Method

### Study population and design

This descriptive study was carried out at traditional batik work places in all 5 districts in Yogyakarta Province including Bantul, Kulon Progo, Sleman, Gunung Kidul, and Yogyakarta City. We went to each district’s center of the traditional batik home industry to enroll participants for this study. We approached all (n = 222) of the workers in the production process from all workplaces. All of them voluntarily agreed to be involved and were enrolled in this study.

### Questionnaire and dermatological examination

Interviews to gain information related to the demographic data, exposure, and occupational skin diseases were conducted using the Nordic Occupational Skin Questionnaire (NOSQ-2002/LONG), translated into Indonesian, and with modifications based on the specific conditions in the traditional batik industry. Interviews and dermatological examinations were carried out by a team of dermatologists supervised by dermatologists with expertise in contact and occupational dermatitis. An interviewer read out the questions to a worker and recorded the answers in the provided column in the questionnaire. All respondents were given enough time to understand and if needed, further explanation regarding each question.

To obtain information about the chemicals used in batik production, we checked the labels of its chemical packages. However, not all packages had a label making it difficult to specify the hazards. Therefore, we used the National Institute for Occupational Safety and Health (NIOSH) pocket guide to chemical hazards^9^, Pubchem, and some references to complete the data. An interview was also done with a researcher at the Center of Crafts and Batik, Ministry of Trade and Industry, the Republic of Indonesia in Yogyakarta, who provided information about each step in the traditional batik manufacturing process, along with the chemicals that were used.

### Ethical considerations

This study received ethical approval from the Medical and Health Research Ethics Committee of the Faculty of Medicine Universitas Gadjah Mada and all research protocols have been performed in accordance with the Declaration of Helsinki. Written informed consent was obtained from all subjects for the study participation in addition to the publication of data including identifying information or images.

## Results

### Working steps and the chemicals involved

**Figure 1:**
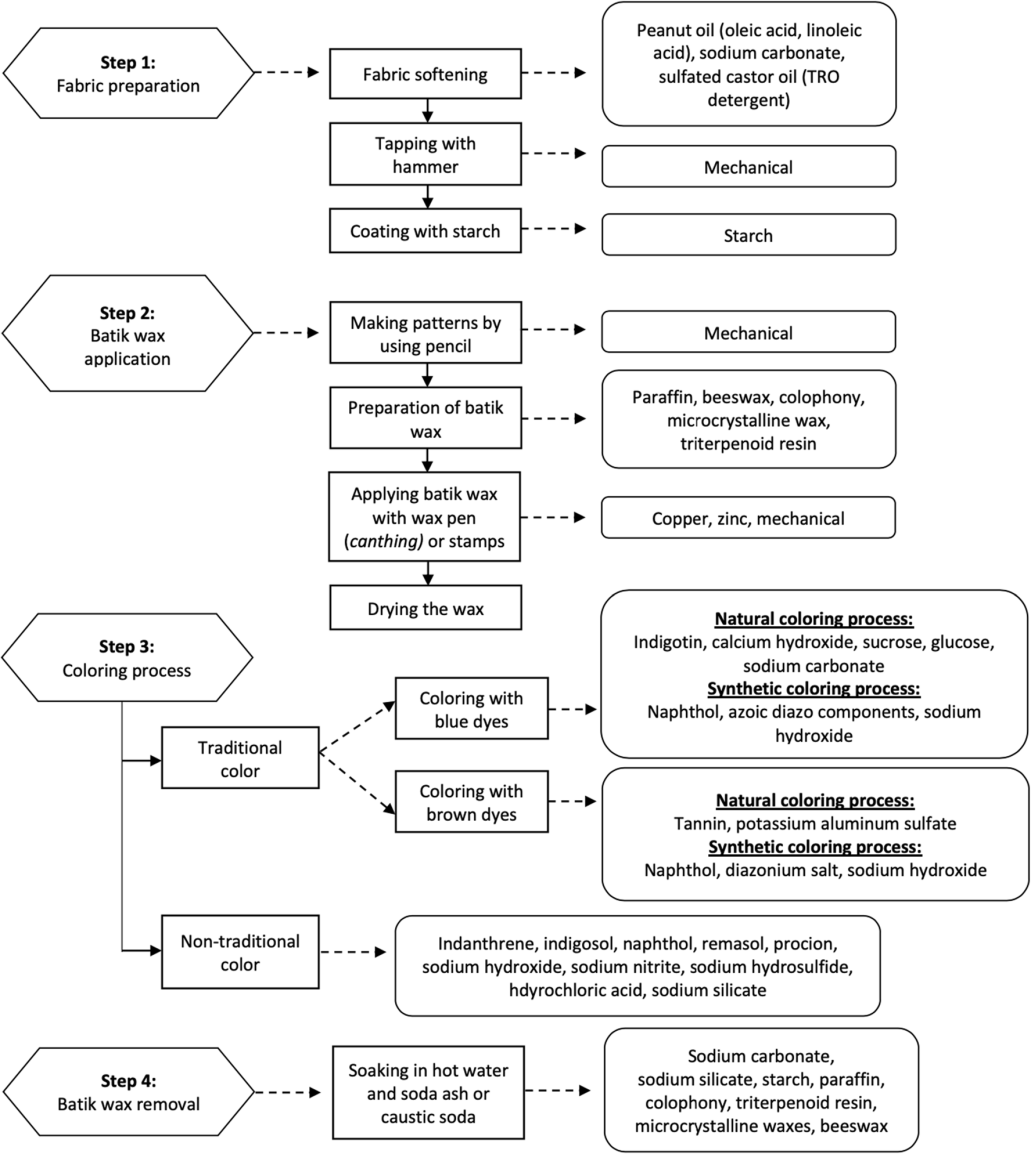
Flowchart of the traditional batik manufacturing process and the skin exposure in each step.

The traditional batik manufacturing process is divided into 4 working steps: (1) fabric preparation; (2) application of batik wax either with a wax pen *(canthing)* or batik pattern stamp on the fabric; (3) coloring/dyeing; (4) removal of the batik wax. All chemicals the workers are exposed to are shown in Fig. 1. Below is an explanation regarding each step.

#### Mori fabric preparation

Natural fibers such as cellulose from plant sources and proteins including wool and silk from animal sources are the preferred materials for making the fabric because of their ability to absorb the wax that is applied in the dye resisting process. Furthermore, these materials do not require a high temperature to be colored, therefore, batik wax does not melt during the dyeing process. Cotton is the most commonly used fabric material. To remove starches, lime, chalk, and other materials and to get a softer cloth, the fabric used for batik is repeatedly washed in water, peanut oil, potassium aluminum sulfate (alum), sodium carbonate, and turkey red oil/TRO detergent (sulfated castor oil) before the application of wax.

However, this step of fabric preparation is no longer done by the traditional batik manufacturers in Yogyakarta, including those in this study. Instead, the fabrics are purchased from suppliers. There are areas in Indonesia where the workers still perform this step.

#### Batik wax application over the fabric either by a wax pen or batik pattern stamp

**Figure 2:**
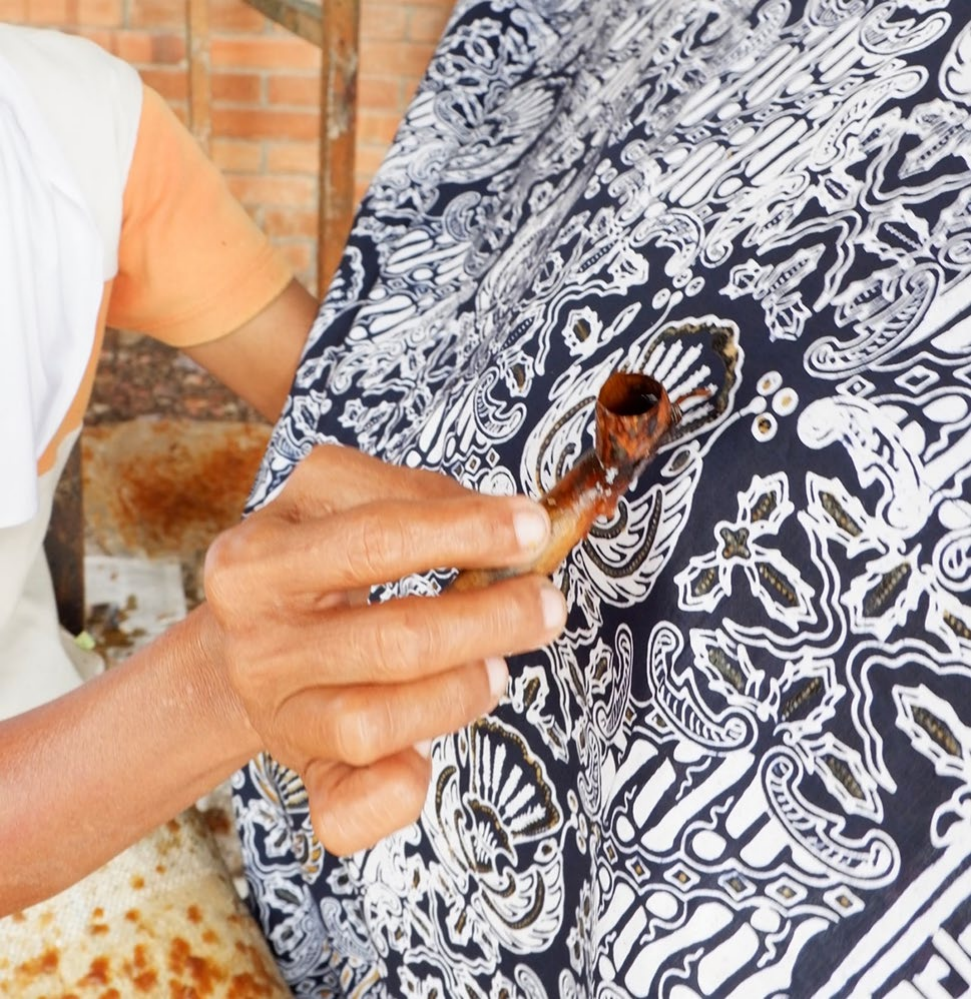
Applicating batik wax with a wax pen.

**Figure 3:**
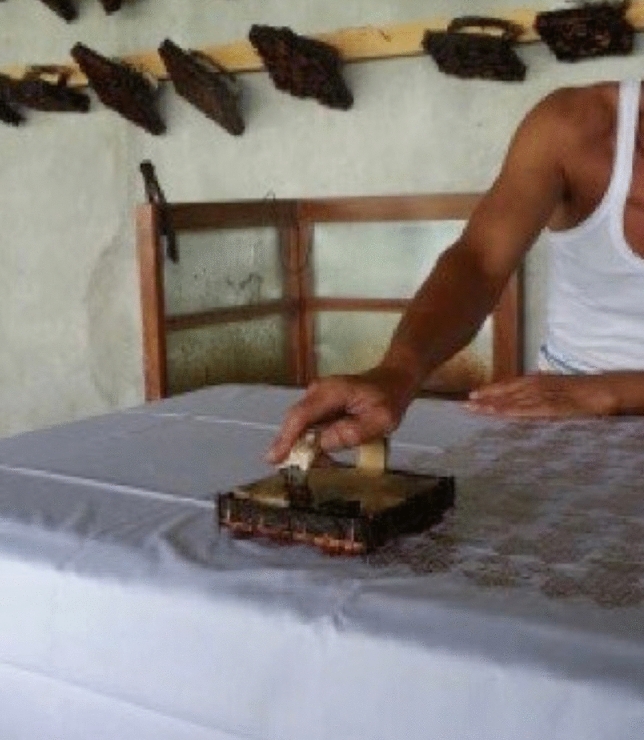
Application of batik wax with a copper stamp.

**Figure 4:**
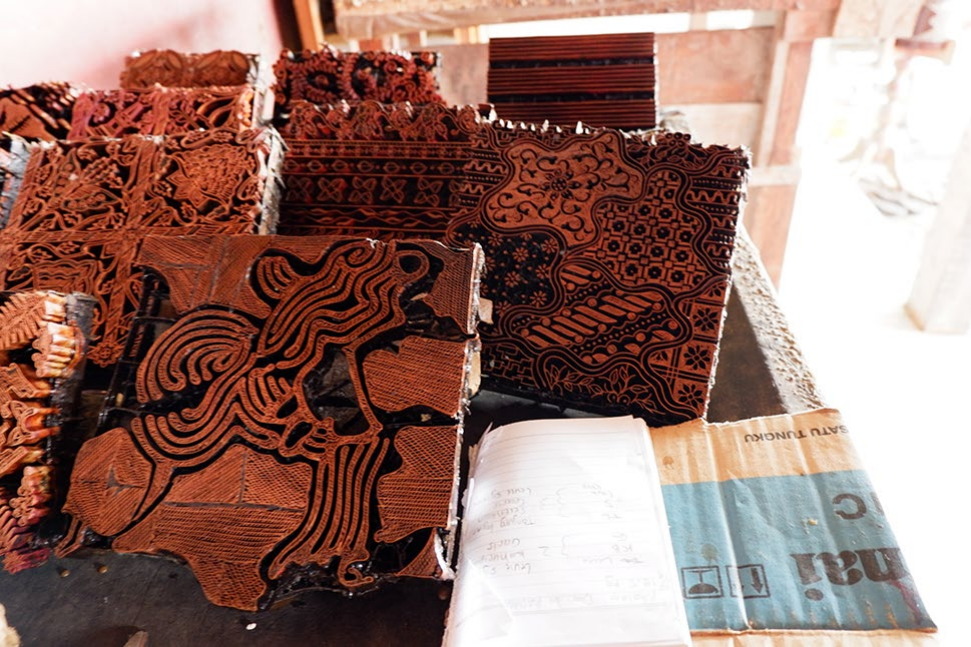
Copper stamps.

The batik pattern is outlined directly on the surface of the fabric by using a pencil, followed by the application of batik wax. This application utilizes a Javanese special wax pen called *Canthing* which consists of a copper container and its bamboo handle of 11 cm, with various sizes of spouts (See Fig. 2). Solid wax is heated on a stove resulting in melted wax which can be put into the container of the wax pen. Melted wax in the container will flow out in a thin stream through a curved spout. Batik stamps are also widely used because they are more practical (See Figs. 3 and 4). Batik stamps are mostly made from Copper although a combination of copper and zinc is sometimes used as well.

**Figure 5:**
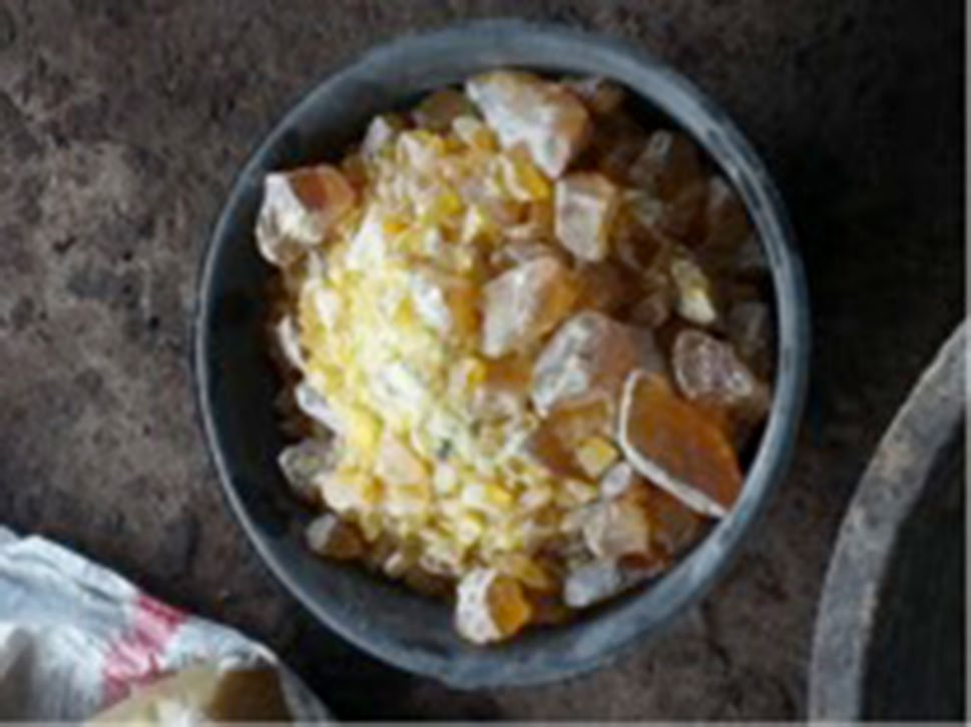
Resin colophony.

Traditional batik involves different kinds and qualities of wax. Generally, batik wax is based on paraffin and contains a mixture of beeswax, microcrystalline wax, colophony, and triterpenoid resin. (See Fig. 5) Beeswax is used for its malleability, while petroleum-based paraffin, including white, yellow, and black paraffin, is used for its friability. Resins can be added to increase adhesiveness and animal fats to create greater liquidity.

#### Coloring/dyeing

The usual Javanese traditional batik uses natural ingredients, with blue, brown, black, and beige as its primary colors. The blue color is derived from Indigofera leaves extracts (indigotin), where the cloth needs to be immersed in the dyebath of indigotin (insoluble), along with its reducing agent and alkaline solution, i.e soda ash. Brown dyes are usually extracted from naturally occurring plants, especially those rich in flavonoids and tannins^10^. *Soga* is a general term that is applied to many types of natural dyeing plants that produce brown colors, such as *Cudriana javanensis*, *Cerios candolleana*, and *Peltophorum pterocarpum*. Dark red is naturally made from the leaves of *Morinda Citrifolia.* For natural dyes, several additives are needed to provide color fixation, such as calcium hydroxide (lime), potassium aluminum sulfate (alum), and iron (II) sulfate (copperas). As a water-insoluble product, indigo needs a reduction agent such as a sodium hydrosulfite (Na_2_SO_4_) solution to produce a water-soluble product known as leuco-indigo.

**Figure 6:**
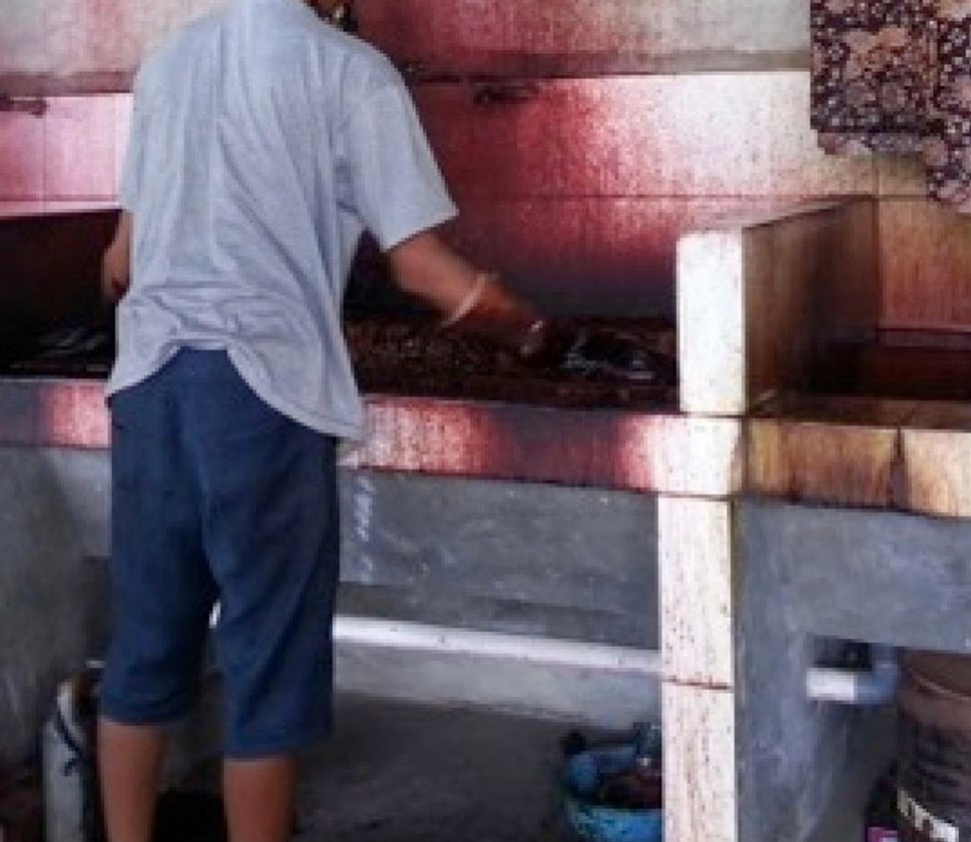
Coloring process.

For practical reasons, synthetic dyes such as synthetic indigo, synthetic soga, naphthol, remasol, procion, indanthrene, and indigosol are nowadays more widely used. All coloring agents are then mixed with auxiliary chemicals to get the desired color. Auxiliary chemicals include sodium silicate, caustic soda, sulfated castor oil, sulfuric acid, sodium hydrosulfite, and sodium nitrite^3,7^. After initial waxing, the fabric is then immersed in a large concrete or clay vase for the first dye bath. To obtain a darker color, sometimes numerous repetitions, or a longer duration of immersion, is required^11,12^ (See Fig. 6).

#### Wax removal

After coloring the fabric, batik wax needs to be removed by using hot water, sodium silicate, sodium carbonate, and starch.

### Characteristics of the chemicals used in each stage

Among all the chemicals exposed to batik home industry workers, some substances act as irritants and/or contact allergens, putting the workers at risk of developing OCD. Characteristics of the chemicals used in this study were assembled by using MSDS, NIOSH, Pubchem, and reference books. In Table 1, this classification of chemicals to which workers are exposed as either an irritant or contact allergen (or both) is summarized.

**Table 1:** Characteristics of the chemicals involved in the batik manufacturing process.

Working process	Chemical used	CAS number	Characteristic
**Step 1: Fabric preparation**			
Soaking	Oleic acid	112-80-1	Irritant
	Linoleic acid	60-33-3	Irritant
	Sodium carbonate/soda ash	497-19-8	Irritant
	Sulfated castor oil/turkey red oil (TRO)	8002-33-3	Irritant
Starch coating	Starch	9005-25-8	Irritant
**Step 2: Batik wax application**			
	Paraffin wax	8002-74-2	Allergen and irritant
	Colophony (resin colophonium)	8050-09-7	Allergen and irritant
	Triterpenoid resin	464-92-6	Irritant
	Microcrystalline waxes	63231-60-7	Irritant
	Beeswax	8012-89-3	Irritant
	Copper	7440-50-8	Allergen
	Zinc	7440-66-6	Irritant
**Step 3: Coloring process**			
Natural blue dyes and additives	Indigotin	68651-46-7	Irritant
	Calcium hydroxide	1305-62-0	Irritant
	Sucrose	57-50-1	Irritant
	Glucose	50-99-7	Irritant
	Sodium carbonate/soda ash	497-19-8	Irritant
Synthetic blue dyes and additives	Naphthol AS-BO	132-68-3	Irritant
	Azoic Diazo Component 48 (fast blue B Salt)	14263-94-6	Irritant
	Azoic diazo component 109 (fast black B base)	6369-04-6	Irritant
	Sodium hydroxide	1310-73-2	Irritant
Natural brown dyes and additives	Tannin/tannic acid	1401-55-4	Irritant
	Potassium aluminum sulfate	7784-24-9	Irritant
	Calcium hydroxide/limewater	1305-62-0	Irritant
	Iron (II) sulfate/*tunjung*	7720-78-7	Irritant
Synthetic brown dyes and additives	Naphthol AS-G	91-96-3	Irritant
	Naphthol AS-LB	132-61-6	Irritant
	Azoic diazo component 5 (Fast Red B Base)	97-52-9	Irritant
	Azoic diazo Component 48 (fast blue B salt)	14263-94-6	Irritant
	Azoic diazo Component 44 (fast yellow GC base)	95-51-2	Irritant
	Sodium hydroxide	1310-73-2	Irritant
Synthetic non-traditional color	Naphthol AS-G	91-96-3	Irritant
	Naphthol AS	92-77-3	Allergen and irritant
	Reactive orange 107 (Remazol golden Yellow RNL)	90597-79-8	Allergen and irritant
	Reactive black 5 (Remazol black B)	17095-24-8	Allergen and irritant
	Reactive blue 21 (Remazol turquoise Blue B)	12236-86-1	Allergen and irritant
	Reactive red 198 (Remazol Red RB 133)	145017-98-7	Irritant
	Reactive yellow 42 (Remazol yellow FG)	12226-63-0	Irritant
	Reactive brown 18 (Remazol brown GR)	12225-73-9	Irritant
	Reactive violet 5 (Remazol brilliant Violet 5R)	12226-38-9	Allergen and irritant
	Reactive red 238	173995-81-8	Allergen and irritant
	Reactive red 228	140876-11-5	Allergen and irritant
	Reactive red 123	61969-31-1	Allergen and irritant
	Procion MX 032 carmine red	N/A	Irritant
	Procion MX 010 bright golden yellow	N/A	Irritant
	Procion MX 068 turqoise	N/A	Allergen and irritant
	Brown indigosol (Vat brown 5)	3989-75-1	Irritant
	Indanthrene	81-77-6	Irritant
	Hydrochloric acid	7647-01-0	Allergen and irritant
	Sodium nitrite	7632-00-0	Irritant
	Sodium hydrosulfide	16,721-80-5	Irritant
	Sodium carbonate/soda ash	497-19-8	Irritant
	Sodium silicate	6834-92-0	Irritant
	Sodium hydroxide/caustic soda	1310-73-2	Irritant
**Step 4: Batik wax removal**			
	Sodium carbonate/soda ash	497-19-8	Irritant
	Sodium silicate	6834-92-0	Irritant
	Paraffin wax	8002-74-2	Allergen and irritant
	Starch	9005-25-8	Irritant
	Colophony (resin colophonium)	8050-09-7	Allergen and irritant
	Triterpenoid resin	464-92-6	Irritant
	Microcrystalline waxes	63231-60-7	Irritant
	Beeswax	8012-89-3	Irritant

N/A: Not Available.

### Subject characteristics

Two hundred and twenty-two workers (62.2% female) were enrolled in this study, with a median age of 41 years (range 18–80 years). The majority of the workers (86.5%) had been working as batik artisans for less than 10 years, and 61.7% of workers work less than 8 h daily. As many workers did more than just one step in the batik manufacturing process, the study categorized all steps into 3 processes of work: dry-process (steps 1 and 2); wet-process (steps 3 and 4), and all processes. Of all subjects, 58.6% performed only the dry process, 26.1% only the wet process, and 15.3% performed all processes. A summary of subject characteristics can be seen in Table 2.

**Table 2:** Subject characteristics and prevalence of OCD.

Characteristic	Subjects (%)	Subjects who had OCD [N (%)]
All subjects	222 (100)	23 (10.4)
**Gender**		
Male	84 (37.8)	19 (22.6)
Female	138 (62.2)	4 (2.9)
**Age (years)**		
18–40	106 (47.7)	10 (9.4)
41–60	102 (45.9)	11 (10.8)
≥ 61	14 (6.3)	2 (14.3)
**History of atopic dermatitis**		
Yes	80 (36)	13 (16.3)
No	142 (64)	10 (7.0)
**Working duration per day (hours)**		
≥ 8	85 (38.3)	11 (12.9)
< 8	137 (61.7)	12 (8.8)
**Length of work experience as batik artisan (years)**		
≥ 10	30 (13.5)	3 (10.0)
< 10	192 (86.5)	20 (10.4)
**Working stage**		
All processes	34 (15.3)	4 (11.8)
Wet process (step 3 and 4)	58 (26.1)	18 (31.0)
Dry process (step 1 and 2)	130 (58.6)	1 (0.8)
**The use of PPE**		
Not always	168 (75.7)	15 (8.9)
Always	54( 24.3)	8 (14.8)

### Work safety standards and the use of personal protective equipment (PPE)

During the workplace observation, we found that not all workplace safety standards, especially the use of PPE, were obeyed by the subjects. Only one-fourth of the workers used PPE regularly. This regular usage of PPE (especially protective gloves) was only observed during the coloring process and batik wax removal and the regular safety boots usage by < 50% of the workers. Table 3 provides a summary of the use of PPE during workplace observation in this study. The recommended PPE type for handling specific chemicals was derived from the related MSDS.

**Table 3:** Personal Protective Equipment observation in the workplace.

Working area	Potential hazards	PPE needed	PPE availability in the workplace	Workplace observation
Fabric preparation	Exposure to chemical dust and textile fibers	Gloves Masks	–	This step was not performed at our study sites
Batik wax application	Thermal Injury Exposure to chemical dust and smoke from the stove which was used for making the *malam*/ batik wax Exposure to malam ingredients containing irritants and/or allergens	Gloves Mask	Gloves	None of the workers used gloves during the application of *malam* wax
Coloring process	Exposure to coloring agents which may act as irritants and/or allergens Exposure to auxiliary chemicals containing strong acid or alkali	Gloves Mask Apron Goggles Safety boots	Gloves Safety boots	Gloves were used by all workers during coloring process Safety boots were used only by < 50% of the workers
Batik wax removal	Soaking in hot water may result in thermal injury Other chemicals may act as irritants and/or allergens	Gloves Mask Apron Goggles Safety boots	Gloves	Gloves were used by the workers only while in contact with hot water Safety boots were used only by < 50% of the workers

**Figure 7:**
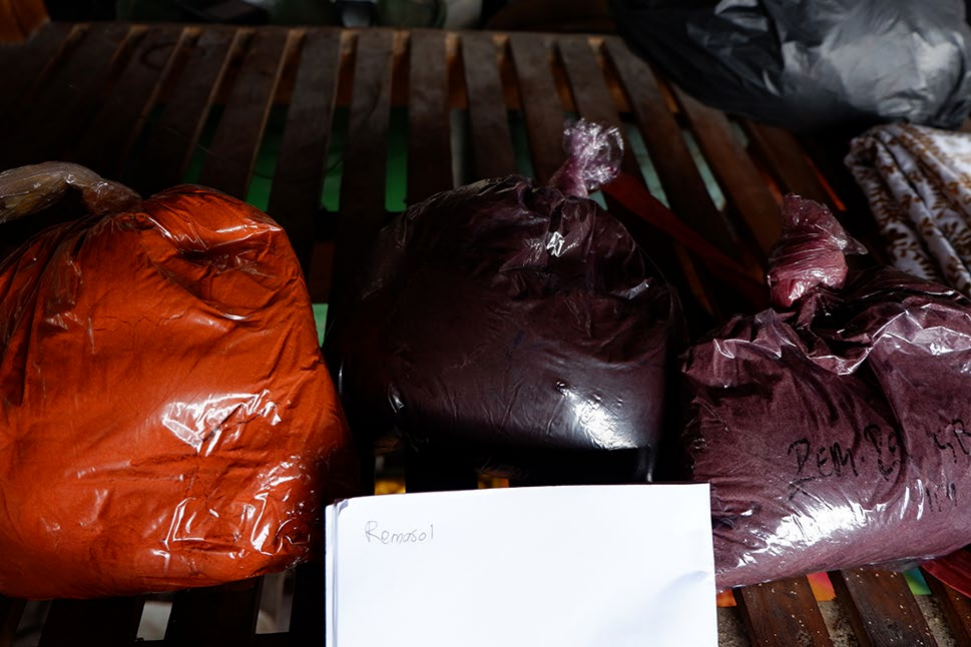
Dye packaging.

Aside from that, we found that some processes were done close to where raw food materials were handled so liquid materials containing dye and batik wax can splash into food containers. Certain dyeing materials were bought in a package with no label, including MSDS and mixing instructions. (See Fig. 7).

### Prevalence of occupational skin diseases and their characteristics

In 222 subjects, we found 61 subjects (27.5%) with occupational skin diseases (OSD), with OCD being the most prevalent OSD (23/61 subjects; 37.7%) and comprised 10.4% of all subjects (23/222 subjects; 10.4%) (Table 2). Occupational irritant contact dermatitis (OICD) and occupational allergic contact dermatitis (OACD) were observed in 16 subjects and 7 subjects respectively^8^. The most frequent locations for OCD in this study include back of the hand (n = 23, 100%), palm (n = 15, 65.2%), sole and upper surface of the foot (n = 11, 47.8%), and lower arm (n = 5, 21.7%). Besides OCD, the most commonly encountered OSD were callus, miliaria, nail disorder, and pityriasis versicolor with prevalences of 9.0%, 6.3%, 5.9%, and 5.9%, respectively (Table 4).

**Table 4:** Occupational skin diseases in batik workers.

Type of OSD	N (%)^a^
Occupational contact dermatitis	23 (10.4)
Irritant contact dermatitis	16 (7.2)
Allergic contact dermatitis	7 (3.2)
Callus	20 (9.0)
Miliaria	14 (6.3)
Nail problems	13 (5.9)
*Pityriasis versicolor*	13 (5.9)
Dermatophyta infection	11 (4.9)
*Pitted keratolysis*	8 (3.6)
Combustio	4 (1.8)

^a^subject can have more than one occupational skin diseases.

## Discussion

There are very little data on health conditions among traditional batik manufacturing workers since this industry is not as widespread as the mass clothing industry. Despite this limitation, several studies have been conducted in Indonesia in which the health conditions among traditional batik manufacturing workers were observed. Results from this study are representative of other traditional batik manufacturers since the processes involved are not very different from each other.

### Prevalence of occupational skin diseases among batik manufacturing workers in Indonesia

In this study, 61 out of 222 (27.5%) subjects had an occupational skin disease (OSD). This number was quite similar to the prevalence of 28.1% in the study performed by Kusbandono et al. on traditional batik manufacturing workers^7^. Soebono et al. found a similar result, with a prevalence of OSD among traditional batik manufacturing workers of 26.9%^12^. Prevalence of OSD as measured by performing a dermatological examination would most likely have been higher than by self-reported measurements, as has been described in a study by Febriana et al. in which fewer workers in a shoe manufacturing factory reported current skin complaints (8.5%) by questionnaire than found by dermatological examination (29.0% OSD)^13^.This might be due to several reasons: (a) they had grown accustomed to their disease, (b) feeling afraid of losing their job by admitting the disease; (c) different levels of education.

Generally, OCD is the most common type of OSD reported in many industries in developed countries (70–90%)^14–16^. In our study, OCD comprised 37.7% of all OSD with a prevalence of 10.4% (n = 23/222) of all subjects. Previous studies by Kusbandono et al. and Soebono et al. showed a slightly higher percentage of OCD among OSD (41,6% and 51%, respectively) in batik workers^7,12^. This finding might indicate that OCD is still the mainstay dermatological problem after decades in the traditional batik industry. Our research team also performed patch tests, which had not been done in other previous studies, by using European baseline and Textile series (Chemotechnique Diagnostics, Vellinge, Sweden) along with additional allergens that are specifically involved in the batik industry setting (published separately)^8^. Our study found that reactive red 123 dye is the largest sensitizer with relevant positive patch test results among the workers employed in the coloring process, followed by reactive red 238, reactive blue 21, and reactive violet 5. Paraffin and colophonium were also positively patch tested in some workers employed in the wax application and removal processes^8^.

Other common occupational skin disorders in this study were callus (9.01%), miliaria (6.3%), nail problems (5.9%), and pityriasis versicolor (5.9%), slightly different from the study by Kusbandono et al.: callus (16.9%), occupational burns (14.6%), tinea pedis (13.5%), and pitted keratolysis (12.3%)^7^. The difference in skin disease variations might be influenced by different environmental factors in occupational settings, but skin diseases such as miliaria, pityriasis versicolor, tinea pedis, and pitted keratolysis are more commonly encountered in humid and wet areas such as the batik workplace^4^.

### Work safety standards and PPE usage

In this study, inconvenience from using gloves at work led to the low usage of this equipment by batik workers, especially during the application of batik wax. This result is similar to a previous study conducted in Semarang, Central Java, Indonesia, which reported that the majority (61.8%) of batik workers did not use gloves and masks while working; the main reason was the inconvenient feeling^17^. Unfortunately, in this study, despite the workers routinely using protective gloves for the coloring process and wax removal, some of the available gloves at the workplace were already worn out or impaired. Some workers also felt the gloves were too loose due to inappropriate size. All of these might lead to the leakage of water and chemical hazards into the gloves and cause skin damage from an occlusive contact^18^.

Several factors should be considered in the selection of gloves in the work area, such as the material of gloves, different hand sizes of employees, and the cuff length^19^. In the batik manufacturing process, the material of gloves should have great dexterity due to its repetitive detailing process, especially during the application of batik wax. A longer cuff length is necessary for immersion activities, such as the coloring process and wax removal, to protect both the hand and forearm from liquids seeping into gloves. Varied sizes of gloves also should be provided to deal with the different hand sizes of the workers. Currently, based on workplace observation, polyvinyl chloride (PVC) is the most commonly used glove material due to its low cost and high protection against various chemicals in batik processing^20^. However, low compliance with PVC gloves usage among the workers is mainly due to the inconvenience while using them and the subsequent lower work quality results. Therefore, further study is needed to evaluate and determine the most convenient protective gloves material to improve the compliance of using PPE during work.

## Conclusion

The traditional batik manufacturing process exposes the worker to various physical and chemical hazards, resulting in a high prevalence of occupational skin diseases, especially contact dermatitis among employees. Inappropriate use of PPE, including the low obedience to use the complete recommended equipment, and the use of impaired or incorrect size gloves at work may contribute to worsening such conditions. Therefore, an improvement in health and safety regulation at work in the traditional batik industry is needed.

## Supplementary Information

Supplementary Information.

## Data Availability

The datasets used and/or analyzed during the current study are available from the corresponding author on reasonable request.
